# Supplementary material for: Exploring Genomic Biomarkers for Pembrolizumab Response: A Real-World Approach and Patient Similarity Network Analysis Reveal DNA Response and Repair Gene Mutations as a Signature
Source: Cancers (Basel). 2024 Nov 26;16(23):3955. doi: 10.3390/cancers16233955 (PMC11639826; doi:10.3390/cancers16233955)
Supplement: Supplementary file 1 [file cancers-16-03955-s001.zip › Table S1a, S1b and S1c.pdf]

Table S1a. Baseline molecular characteristics of the real-world cohort by smoking status.

Oncogenic Driver	S-pts N = 111<br>n (%)	NS-pts N = 31<br>n (%)
KRAS G12C	20(18)	3(12)
EGFR	0(0)	0(0)
BRAF V600E	2(2)	1(3)
MET	3(3)	3(10)
HER2	4(4)	1(3)
RET	2(2)	1(3)
ROS1	0(0)	0(0)
ALK	0(0)	0(0)
PIK3CA	1(1)	1(3)
BRAF non V600E	4(4)	2(6)
EGFR ex20	1(1)	2(6)
KRAS non G12C	20(18)	2(6)

Abbreviations: S-pts, smoking patient (smokers or former smokers); NS-pts, never-smoking patients).

Table S1b. Table list of analyzed genes.

ABL1 ACVR1B AKT1 AKT2 AKT3 ALK ALOX12B AMER1 (FAM123B) APC AR ARAF
ARFRP1 ARID1A ASXL1 ATM ATR ATRX AURKA AURKB AXIN1 AXL BAP1 BARD1
BCL2 BCL2L1 BCL2L2 BCL6 BCOR BCORL1 BRAF BRCA1 BRCA2 BRD4 BRIP1 BTG1
BTG2 BTK C11orf30 (EMSY) C17orf39 (GID4) CALR CARD11 CASP8 CBFB CBL CCND1
CCND2 CCND3 CCNE1 CD22 CD274 (PD-L1) CD70 CD79A CD79B CDC73 CDH1 CDK12
CDK4 CDK6 CDK8 CDKN1A CDKN1B CDKN2A CDKN2B CDKN2C CEBPA CHEK1
CHEK2 CIC CREBBP CRKL CSF1R CSF3R CTCF CTNNA1 CTNNB1 CUL3 CUL4A CXCR4
CYP17A1 DAXX DDR1 DDR2 DIS3 DNMT3A DOT1L EED EGFR EP300 EPHA3 EPHB1
EPHB4 ERBB2 ERBB3 ERBB4 ERCC4 ERG ERRFI1 ESR1 EZH2 FAM46C FANCA FANCC
FANCG FANCL FAS FBXW7 FGF10 FGF12 FGF14 FGF19 FGF23 FGF3 FGF4 FGF6 FGFR1
FGFR2 FGFR3 FGFR4 FH FLCN FLT1 FLT3 FOXL2 FUBP1 GABRA6 GATA3 GATA4
GATA6 GNA11 GNA13 GNAQ GNAS GRM3 GSK3B H3F3A HDAC1 HGF HNF1A HRAS
HSD3B1 ID3 IDH1 IDH2 IGF1R IKBKE IKZF1 INPP4B IRF2 IRF4 IRS2 JAK1 JAK2 JAK3
JUN KDM5A KDM5C KDM6A KDR KEAP1 KEL KIT KLHL6 KMT2A (MLL) KMT2D
(MLL2) KRAS LTK LYN MAF MAP2K1 (MEK1) MAP2K2 (MEK2) MAP2K4 MAP3K1
MAP3K13 MAPK1 MCL1 MDM2 MDM4 MED12 MEF2B MEN1 MERTK MET MITF MKNK1
MLH1 MPL MRE11A MSH2 MSH3 MSH6 MST1R MTAP MTOR MUTYH MYC MYCL
(MYCL1) MYCN MYD88 NBN NF1 NF2 NFE2L2 NFKBIA NKX2-1 NOTCH1 NOTCH2
NOTCH3 NPM1 NRAS NSD3 (WHSC1L1) NT5C2 NTRK1 NTRK2 NTRK3 P2RY8 PALB2
PARK2 PARP1 PARP2 PARP3 PAX5 PBRM1 PDCD1 (PD-1) PDCD1LG2 (PD-L2) PDGFRA
PDGFRB PDK1 PIK3C2B PIK3C2G PIK3CA PIK3CB PIK3R1 PIM1 PMS2 POLD1 POLE
PPARG PPP2R1A PPP2R2A PRDM1 PRKAR1A PRKCI PTCH1 PTEN PTPN11 PTPRO QKI
RAC1 RAD21 RAD51 RAD51B RAD51C RAD51D RAD52 RAD54L RAF1 RARA RB1 RBM10
REL RET RICTOR RNF43 ROS1 RPTOR SDHA SDHB SDHC SDHD SETD2 SF3B1 SGK1
SMAD2 SMAD4 SMARCA4 SMARCB1 SMO SNCAIP SOCS1 SOX2 SOX9 SPEN SPOP SRC
STAG2 STAT3 STK11 SUFU SYK TBX3 TEK TET2 TGFBR2 TIPARP TNFAIP3 TNFRSF14
TP53 TSC1 TSC2 TYRO3 U2AF1 VEGFA VHL WHSC1 WT1 XPO1 XRCC2 ZNF217 ZNF703

Table S1c. Table list of pathway member genes.

Pathway	Description	Genes	References
Cell Cycle	Regulation of mitotic cell cycle progression involving a signaling cascade of cyclins and cyclin-dependent kinases as well as a number of regulatory checkpoints.	CDKN1A/B, CDKN2A/B/C, CCND1/2/3, CCNE1, CDK2/4/6, RB1, E2F1/3.	(Malumbres and Barbacid, 2009)
HIPPO	Involved in the control of organ size. Central to this pathway is the regulation of the transcription co-activators YAP/TAZ that promote the transcription of genes involved in cell proliferation.	STK4/3, SAV1, LATS1/2, MO1A/1B, YAP1, TAZ (WWTR1), TEAD1/2/3/4, PTPN14, NF2, TAOK1/2/3, CRB1/2/3, LLGL1/2, HMCN1, SCRIB, HIPK2, FAT1/2/3/4, DCHS1/2, CSNK1E, CSNK1D, AJUBA, LIMD1,WTIP.	(Harvey et al., 2013)
MYC	Involves a number of transcription regulation complexes: MYC-MAX, MAX-MXD, MAX-MGA, and the energy sensing, MondoA-Mlx complex in the regulation of apoptotic response and cell differentiation.	MAX, MGA, MLX, MLXIP, MLXIPL, MNT, MD1/3/4, MXI1, MYC, MYCL, MYCCN.	(Nilsson and Cleveland, 2003)
NOTCH	Pathway involved in cell-cell communication, cell fate. Cleavage of Notch receptors leads to the displacement of a transcription repressor complex on RBPJ (a transcription factor also known as CSL) accompanied by recruitment of an activation complex (including MAMLs) leads to transcription of Notch target genes.	NOTCH1/2/3/4, JAG1/2, EP300, ARRDC1, CNTN, CREBBP, HES1/2/3/4/5, HEY/1/2, HEYL, KAT2B, KDM5A, NOV, NRARP, PSEN1/2, LFNG, ITCH, NCSTN, SPEN, APH1A, FBXW7, FHL1, THBS2, HDAC2, MFAP2, CUL1, RFNG, NCOR1/2, MFAP5, HDAC1, NUMB, MAML3, MFNG, CIR1, CNTN1, MAML1/2, NUMBL, PSENEN, RBPJ, RBPJL, RBX1, SAP30, SKP1, SNW1, CTBP1/2. ADAM10, APH1B, ADAM17, DLK1, DLL1/3/4, DNER, DTX1/2/3/4, DTX3L, EGFL7	(Radtke and Raj, 2003)
NRF2	Involves the regulation of the transcription factor NFE2L2 by KEAP1. NFE2L2 regulates genes with the antioxidant response elements (ARE) that aid in cellular response against oxidative stress thought to aid in cancer chemoresistance.	NFE2L2, KEAP1, CUL3	(Sporn and Liby, 2012)
PI3K	A signaling cascade involving PI3K phosphorylation of AKT	EIF4EBP1,AKT1, AKT2 , AKT3	(Proud, 2013)

	leading to the activation of the mTORC1 complex. The mTORC1 functions as a metabolic sensor and controls protein abundance by affecting processes involved in protein production and RNA translation leading to changes in cell growth and survival.	, AKT1S1, DEPDC5, DEPTOR INPP4B, MAPKAP1, MLST8 , MTOR, NPRL2, NPRL3, PDK1, PIK3CA, PIK3CB, PIK3R1, PIK3R2, PIK3R3, PPP2R1A, PTEN, RHEB, RICTOR, RPTOR, RPS6, RPS6KB1, STK11, TSC1, TSC2	
RTK/RAS	A signaling cascade pathway initiated by activation of RTKs followed signal transduction through Ras then Raf and then MEK family members. This cascade leads to the activation of several transcription factors that regulate processes involving cell proliferation and survival.	ABL1, EGFR, ERBB2, ERBB3 ERBB4, PDGFRA, PDGFRB, MET, FGFR1, FGFR2, FGFR3, FGFR4, FLT3, ALK, RET, ROS1 KIT, IGF1R, NTRK1, NTRK2, NTRK3, SOS1, GRB2, PTPN11, KRAS, HRAS, NRAS, RIT1, ARAF, BRAF, RAF1, RAC1, MAP2K1, MAP2K2, MAPK1, NF1, RASA1, CBL, ERRFI1, CBLB, CBLC, INSR, INSRR, IRS1, SOS2, SHC1, SHC2, SHC3, SHC4, RASGRP1, RASGRP2 RASGRP3, RASGRP4 RAPGEF1, RAPGEF2, RASGRF1, RASGRF2, FNTA, FNTB, RCE1, ICMT, MRAS, PLXNB1, MAPK3, ARHGAP35, RASA2, RASA3, RASAL1, RASAL2, RASAL3, SPRED1, SPRED2, SPRED3, DAB2IP,SHOC2 ,PPP1CA, SCRIB, PIN1, KSR1, KSR2, PEBP1, ERF, PEA15, JAK2, IRS2	(Malumbres and Barbacid, 2003)
TGFβ	A signaling network involved in growth, proliferation, apoptosis, and differentiation involving the activation of TGFβ receptors by the cytokine TGFβ that leads to the activation of gene transcription by SMADs.	SMAD 2/3/4, TGFBR1/2, ACVR2A, ACVR1B	(Massagué, 2008)
TP53	Pathway centered around the regulation of the tumor suppressor TP53, a gene that regulates apoptosis, cell cycle arrest, senescence, and DNA repair.	TP53, CDKN2A, ATM, MDM2/4, CHECK2, RPS6KA3	(Wade et al., 2013)
WNT	Involved in both development and tissue homeostasis. The canonical Wnt pathway involves signal transduction initiated by Wnt ligand binding to Frizzled family receptors leading to the dysregulation of beta-catenin degradation and ultimately, the	CHD8, LEF1, LGR4, LGR5 LRP5, LRP6, LZTR1, NDP, PORCN, RSPO1, SFRP1, SFRP2, SFRP4, SFRP5, SOST, TCF7L1, TLE1, TLE2, TLE3, TLE4, WIF1, ZNRF3, CTNNB1, DVL1, DVL2, DVL3, FRAT1, FRAT2, FZD1, FZD10, FZD2, FZD3, FZD4, FZD5,	(Reya and Clevers, 2005)

	induction of transcription via TCF/LEF transcription factors by beta-catenin.	FZD6, FZD7, FZD8, FZD9, WNT1, WNT10A, WNT10B, WNT11, WNT16, WNT2, WNT3A, WNT4, WNT5A, WNT5B, WNT6, WNT7A, WNT7B, WNT8A, WNT8B, WNT9A, WNT9B, AMER1, APC, AXIN1, AXIN2, DKK1, DKK2, DKK3, DKK4, GSK3B, RNF43, TCF7, TCF7L2, CHD4	
DNA damage repair (DDR)	Cells have evolved a number of pathways that comprise a network of proteins that sense, signal and/or repair DNA, which are collectively referred to as the DNA Damage Response (DDR)	Mismatch Repair:<br>MLH1, MLH3, MSH2, MSH6, PMS1, PMS2<br>DNA Damage Sensing:<br>ATM, ATR, CHEK1, CHEK2<br>Homologous Recombination: BRCA1, BRCA2, RAD21, RAD50, RAD51, RAD51C, RAD51D, RAD52, RAD54B, PALB2, BRIP1, BARD1, BAP1<br>Nucleotide Excision Repair: ERCC1, ERCC2, ERCC3, ERCC4, ERCC5, ERCC6<br>Base excision Repair: XRCC1, XRCC2, XRCC3, XRCC4, XRCC5, XRCC6<br>DNA Polymerase:<br>POLB, POLD1, POLE, POLH, POLQ<br>Fanconi Anemia: FANCA, FANCB, FANCC, FANCD2, FANCE, FANCF, FANCG, FANCI, FANCL, FANCM<br>NEIL1 NEIL2 NEIL3, c11orf30, ARID1A1,	Ricciuti B. et Al.Clin Cancer Res. 2020
Others		DNMT3A, FGF19, FGF4, FGF3,MTAP, GNAS, KEL, ET2, BCL2BL1, FRS2, GLI1, SETD2, SMARCA4, ZNF217, ARFRP1, NSD3, PIM1, TERC, TNFAIP3, VEGFA, ZNF703, BRD4, ASXL1, KDM5C, MLL2, FGF10, PTPRO, DIS3, TET2, HGF, FGF6, FGF23, RBM10, PRKCI, SOX2, FGF12, NFKBIA, NKX2, TBX3, MUTYH, PMS2, U2AF1, PRDM1, CASP8, CDC73, FAM123B, NFKBIA, CRKL, TNFRSF14, VHL, PBRM1, RBM10, PARK2, FUBP1, CDK12, KDM5C, ATRX, BCOR, SOX9, KDM6A, NFKBIA, MCL1, SDHD,	

		DAXX, AURKA, MERTK, AXL, WHSC1L1, BCORL1, PRKCI, FLCN, SRC, KDR, CD170, IKZF1, SGK1, CTNNA1, STAG2, REL, RBM10.	

References

Harvey, K.F., Zhang, X., and Thomas, D.M. (2013). The Hippo pathway and human cancer. Nat. Rev. Cancer *13,* 246–257.

Malumbres, M., and Barbacid, M. (2003). RAS oncogenes: the first 30 years. Nat. Rev. Cancer *3,* 459–465.

Malumbres, M., and Barbacid, M. (2009). Cell cycle, CDKs and cancer: a changing paradigm. Nat. Rev. Cancer *9,* 153–166.

Massagué, J. (2008). TGFbeta in Cancer. Cell *134,* 215–230.

Nilsson, J.A., and Cleveland, J.L. (2003). Myc pathways provoking cell suicide and cancer. Oncogene *22,* 9007–9021.

Proud, C.G. (2013). mTORC1 regulates the efficiency and cellular capacity for protein synthesis. Biochem. Soc. Trans. *41,* 923–926.

Radtke, F., and Raj, K. (2003). The role of Notch in tumorigenesis: oncogene or tumour suppressor? Nat. Rev. Cancer *3,* 756–767.

Reya, T., and Clevers, H. (2005). Wnt signalling in stem cells and cancer. Nature *434,* 843–850.

Sporn, M.B., and Liby, K.T. (2012). NRF2 and cancer: the good, the bad and the importance of context. Nat. Rev. Cancer *12,* 564–571.

Wade, M., Li, Y.-C., and Wahl, G.M. (2013). MDM2, MDMX and p53 in oncogenesis and cancer therapy. Nat. Rev. Cancer *13,* 83–96.

Ricciuti B. et Al. Impact of DNA Damage Response and Repair (DDR) Gene Mutations on Efficacy of PD-(L)1 Immune Checkpoint Inhibition in Non-Small Cell Lung Cancer. Clin Cancer Res. 2020 Aug 1;26(15):4135-4142. doi: 10.1158/1078-0432.CCR-19-3529. Epub 2020 Apr 24. PMID: 32332016.
