# Supplementary figures and images for: Exploring Genomic Biomarkers for Pembrolizumab Response: A Real-World Approach and Patient Similarity Network Analysis Reveal DNA Response and Repair Gene Mutations as a Signature
Source: Cancers (Basel). 2024 Nov 26;16(23):3955. doi: 10.3390/cancers16233955 (PMC11639826; doi:10.3390/cancers16233955)

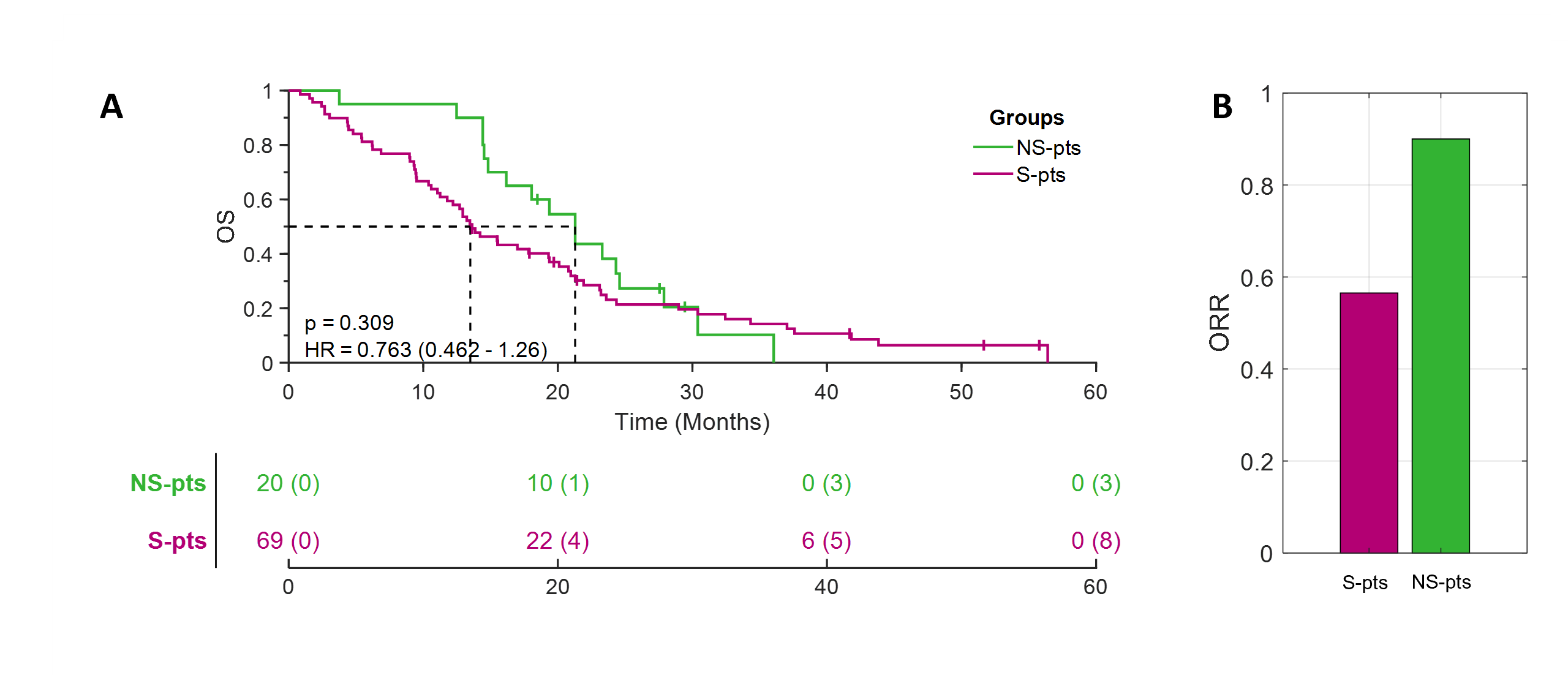

Supplement: Supplementary file 1 [file cancers-16-03955-s001.zip › SI_Figure S1 revised.tif]

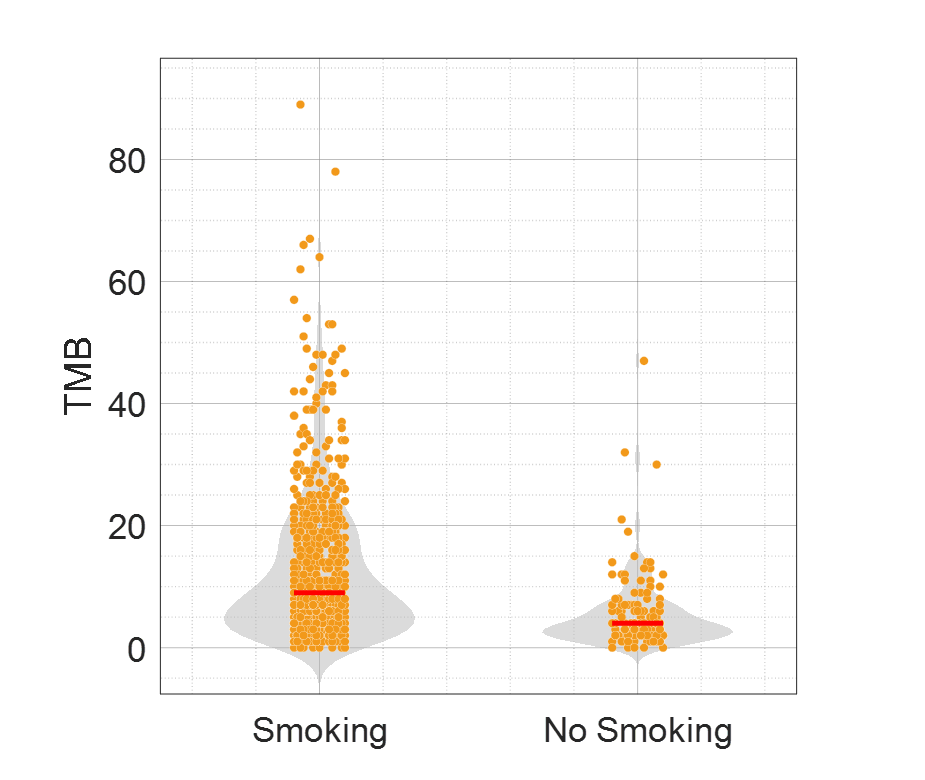

Supplement: Supplementary file 1 [file cancers-16-03955-s001.zip › SI_Figure S2.tif]

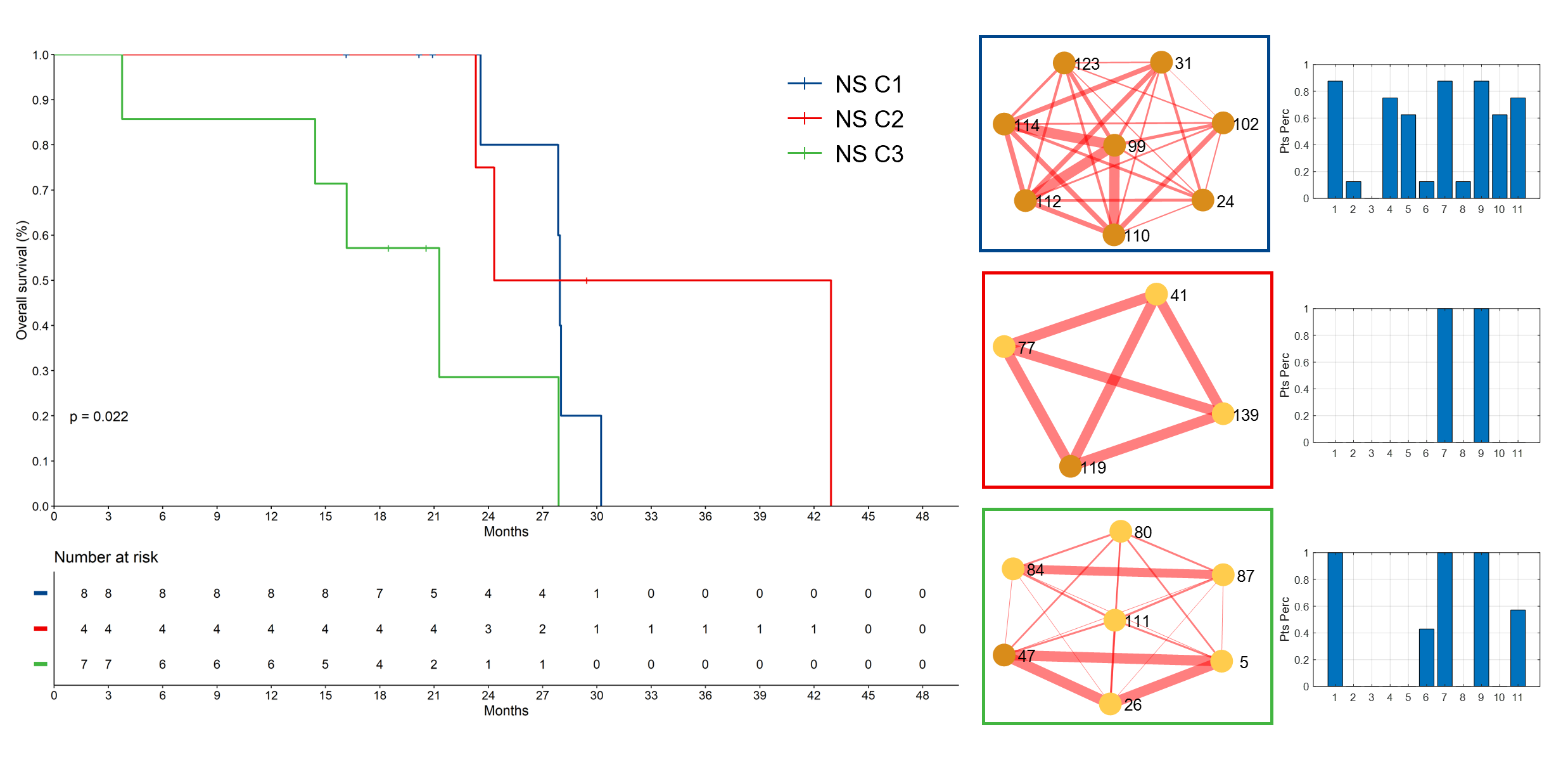

Supplement: Supplementary file 1 [file cancers-16-03955-s001.zip › SI_Figure S3.tif]

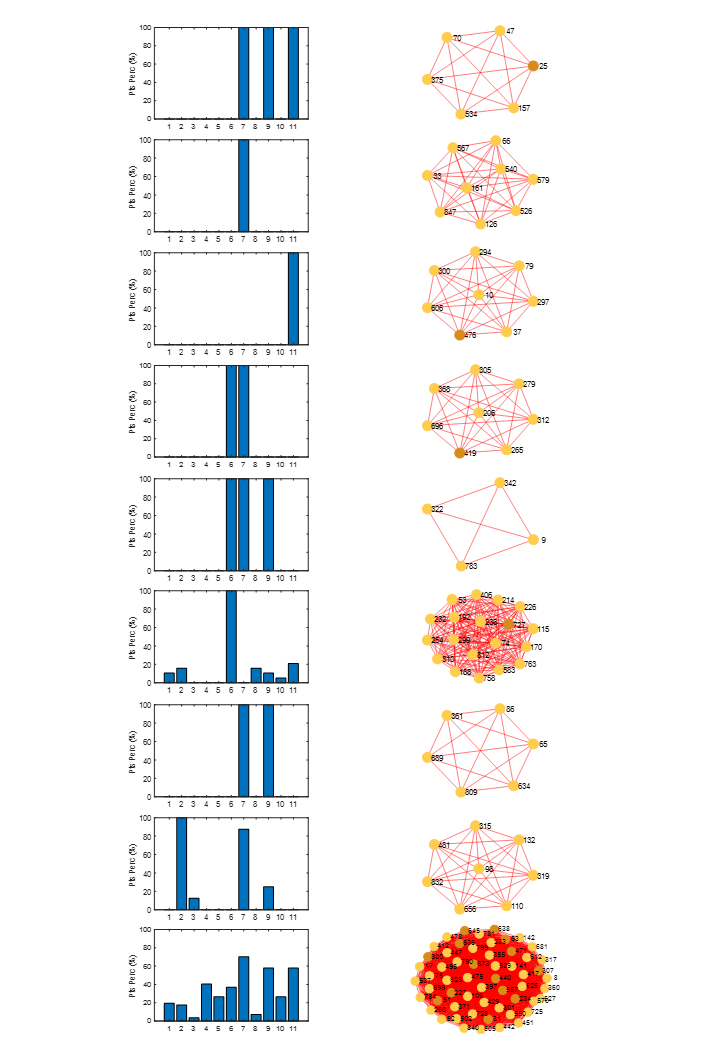

Supplement: Supplementary file 1 [file cancers-16-03955-s001.zip › SI_Figure S4 revised.tif]

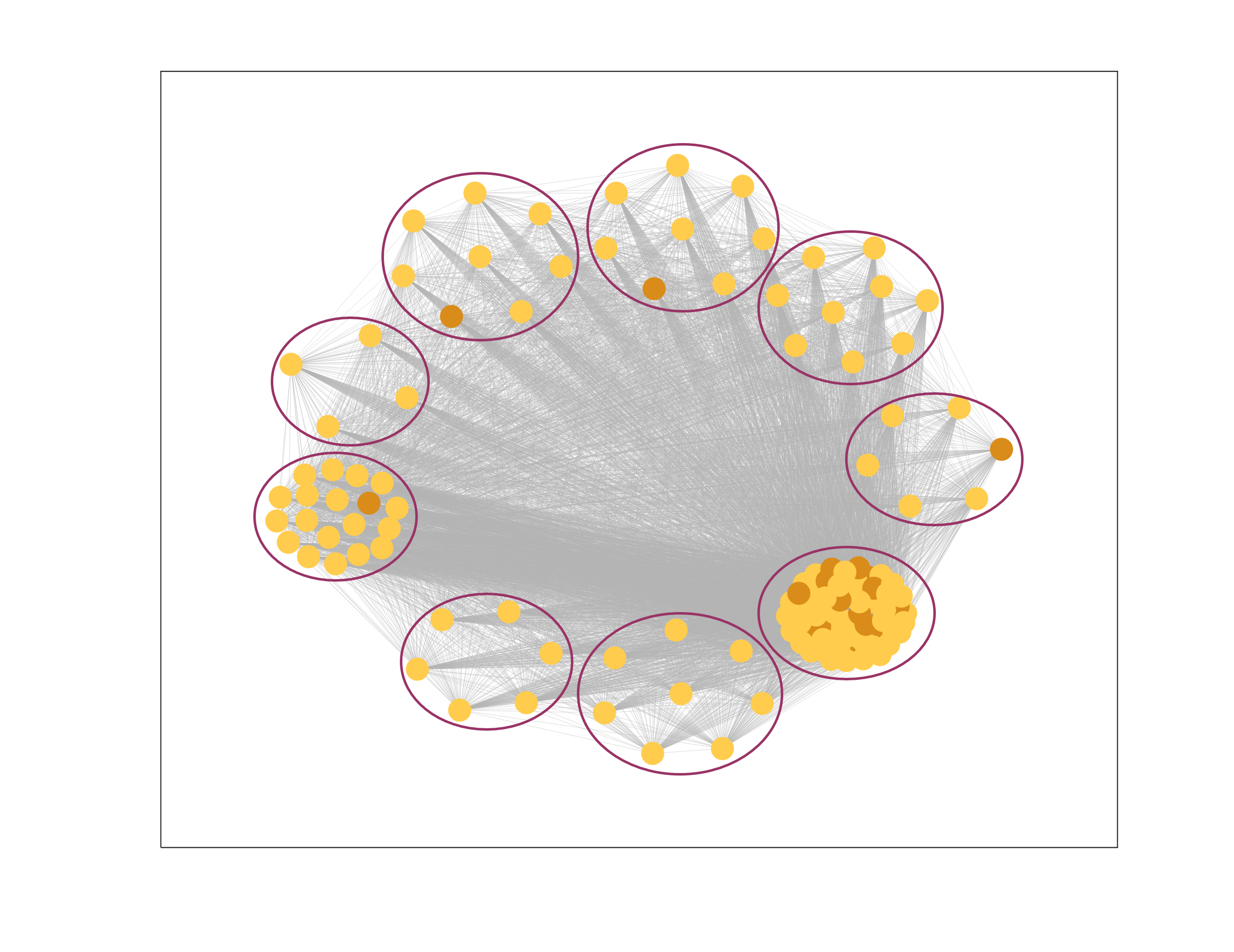

Supplement: Supplementary file 1 [file cancers-16-03955-s001.zip › SI_Figure S5.tif]
